# Periodic Polymerization and the Generation of Polymer Giant Vesicles Autonomously Driven by pH Oscillatory Chemistry

**DOI:** 10.3389/fchem.2021.576349

**Published:** 2021-02-22

**Authors:** Jinshan Guo, Eszter Poros-Tarcali, Juan Pérez-Mercader

**Affiliations:** ^1^ Department of Earth and Planetary Science and Origins of Life Initiative, Harvard University, Cambridge, MA, United States; ^2^ Santa Fe Institute, Santa Fe, NM, United States

**Keywords:** pH oscillator, polymerization, free radicals, vesicles, micelles, amphiphilic block copolymer, redox, self-assembly

## Abstract

Using the radicals generated during pH oscillations, a semibatch pH oscillator is used as the chemical fuel and engine to drive polymerization induced self-assembly (PISA) for the one-pot autonomous synthesis of functional giant vesicles. Vesicles with diameters ranging from sub-micron to ∼5 µm are generated. Radical formation is found to be switched ON/OFF and be autonomously controlled by the pH oscillator itself, inducing a periodic polymerization process. The mechanism underlying these complex processes is studied and compared to conventional (non-oscillatory) initiation by the same redox pair. The pH oscillations along with the continuous increase in salt concentration in the semibatch reactor make the self-assembled objects undergo morphological evolution. This process provides a self-regulated means for the synthesis of soft giant polymersomes and opens the door for new applications of pH oscillators in a variety of contexts, from the exploration of new geochemical scenarios for the origin of life and the autonomous emergence of the necessary free-energy and proton gradients, to the creation of active functional microreactors and programmable release of cargo molecules for pH-responsive materials.

## Introduction

Amphiphilic copolymers can self-assemble into various structures, making them increasingly attractive in the design and construction of functional materials (Discher and Eisenberg, 2002). Giant vesicles (GVs), with a diameter of >1 μm, can also be formed from amphiphilic copolymers (Walde et al., 2010). These giant vesicles are popular protocell models, since they can mimic the essential feature of living systems, the compartmentalization from their environment (Zhang et al., 2012; LoPresti et al., 2009; Rideau et al., 2018). The ubiquitous presence of an interface separating the aqueous contents of the living cell from its external environment is fundamental to extant life on planet Earth and one of its hallmarks. In addition, the *de novo* synthesis of such copolymer-based vesicles from simpler components in a one pot reaction is also possible using the techniques of polymerization induced self-assembly (PISA) and brings closer the realization of autonomous chemical system self-assembly and evolution (D'Agosto et al., 2020; Rieger, 2015; Penfold et al., 2019; Warren and Armes, 2014; Canning et al., 2016; Yeow and Boyer, 2017; Charleux et al., 2012). In PISA, the reversible addition-fragmentation chain transfer (RAFT) polymerization is a popular and basic choice for the synthesis of amphiphilic copolymers with low dispersity (Chiefari et al., 1998; Perrier, 2017). Starting, for example, with some solvophilic macroRAFT agent (or chain transfer agent, CTA), its chain-extension with a solvophobic monomer chain generates living (in the sense of polymer chemistry) amphiphiles. This non-equilibrium process brings about the simultaneous self-assembly of the amphiphiles into collective structures such as micelles, “worms” or vesicles controlled by parameters such as concentration or packing parameter (Israelachvili, 2011) of the generated amphiphiles, pH, temperature, salt concentration, etc., The uncomplicated and simple controllability of the PISA process, make PISA-generated polymersomes excellent candidates for many applications ranging from drug delivery or macro-/nano-reactors, to protocell designs (Cheng and Pérez-Mercader, 2020; Canning et al., 2016; Yeow and Boyer, 2017).

The initiation of RAFT-polymerization requires an external radical source. Besides light induced initiation and thermal initiation, redox chemistry can serve as radical source for RAFT mediated PISA, although the numbers of examples are limited (Yeow and Boyer, 2017; Penfold et al., 2019). Most oscillating chemical reactions involve redox steps and some of these are known to generate free radicals. The Belousov-Zhabotinsky (B-Z) reaction has been reported to initiate the homopolymerization of different monomers (Pojman et al., 1992). The roles of the different free radicals in the B-Z reaction were investigated (Försterling et al., 1990; Venkataraman and Soerensen, 1991; Washington et al., 1999). Recently, the B-Z reaction has been coupled to PISA (Bastakoti and Pérez-Mercader, 2017a; Bastakoti and Pérez-Mercader, 2017b; Bastakoti et al., 2018; Hou et al., 2019; Cheng and Pérez-Mercader, 2020). The second group of oscillatory chemical reactions, which has been successfully applied to initiate RAFT-mediated PISA, was the family of pH oscillators. Specifically, the semibatch BrO_3_^−^−SO_3_^2–^ pH oscillator driven RAFT polymerization of butyl acrylate monomer on macroRAFT agent with poly(ethylene glycol) stabilizing group (Guo et al., 2019). Prior to this work neither the BrO_3_^−^−SO_3_^2–^ pH oscillator, nor any other pH oscillator have been known to be capable to initiate polymerization, although the BrO_3_^−^/SO_3_^2-^ redox pair has been used previously as initiator in free radical homopolymerization (Mukherjee et al., 1966; Liu and Brook, 1998; Liu and Brooks, 1999). The oscillatory chemical reaction in a PISA coupled system goes beyond the role of the radical source. The formed vesicles are also functionalized due to the entrapped active chemical reaction. Furthermore, the intrinsically far-from-equilibrium oscillatory chemical reactions are beneficial in protocell models, hence life cannot exist at a thermodynamically equilibrium and periodicity is known to be fundamental in our living and non-living environment.

In this work our primary aim was to investigate the free radical formation and the initiation of polymerization by the semibatch BrO_3_^−^−SO_3_^2–^ pH oscillator, including the effect of the oscillatory behavior on the polymerization in comparison with conventional (non-oscillatory) redox initiation. The semibatch reactor setup let us maintain the constant supply of the SO_3_^2–^, which is necessary to generate large amplitude pH oscillations and prevents the outwash of the forming polymer (Szántó and Rábai, 2005; Orbán et al., 2015; Poros et al., 2015). As monomer, the hydroxypropyl methacrylate (HPMA) was chosen. HPMA has not been used in any oscillatory chemistry-initiated systems before, despite being one of the few monomers which can be polymerized via aqueous dispersion RAFT polymerization. Furthermore, the PISA of HPMA is extensively studied because it was found to be compatible with various macroRAFT agents, and to be capable to generate all kinds of morphologies of the expected phase diagram (spheres, worms, vesicles, framboidal vesicles, jellyfish) (Warren et al., 2014; Canning et al., 2016).

## Materials and Methods

Poly(ethylene glycol) 4-cyano-4(phenylcarbonothioylthio)pentanoate (PEG-CTA) was synthesized by the esterification reaction between methoxy poly(ethylene glycol) (mPEG, molecular weight = 1900 Da, Fluka), 4-cyano-4-(thiobenzoylthio) pentanoic acid (CTA, Strem Chemicals) with the help of DCC (N,N′-dicylohexylcarbodiimide, Sigma-Aldrich) and DMAP (N,N′-dimethylaminopyridine, Alfa Aesar), according to literature (Szymański and Pérez-Mercader, 2016). Methanol-d4 for NMR tests was purchased from Cambridge Isotope Laboratories, Inc.,. Hydroxypropyl methacrylate (HPMA, mixture of isomers, Alfa Aesar), sodium bromate (NaBrO_3_, Sigma-Aldrich), sodium sulfite (Na_2_SO_3_, anhydrous, Sigma-Aldrich), sulfuric acid (H_2_SO_4_, 10 Normal, Ricca Chemical Company), anhydrous dichloromethane (DCM, Sigma-Aldrich), N,N-dimethylformamide (DMF, HPLC grade, VWR), and lithium bromide (LiBr, anhydrous, 99.99%, VWR) were used without further purification. Sodium sulfite (Na_2_SO_3_) solution was freshly prepared and used on the same day.

The pH change of the aqueous solution in semibatch or batch experiments was monitored by a Benchtop pH/mV Meter (Sper Scientific Direct) equipped with a pH Electrode with BNC + PIN connector (Hanna Instruments), pH values were collected once per second. In semibatch experiments 20 ml of 0.10 M sodium-bromate solution (in some experiments H_2_SO_4_ was added into the sodium bromate solution to lower the initial pH value) was added in a reactor (50 ml), which was thermostated at 25 or 40°C and stirred at 400 rpm. Then a freshly calibrated pH meter was inserted. PEG-CTA (17.3 mg, 8 μmol) and HPMA (72.9–145.9 μl, 520–1,040 μmol) were added into the solution, then the premixed solution of sodium sulfite and sulfuric acid [c(Na_2_SO_3_) = 2.0 M and c(H^+^) = 0.21–0.50 M] was continuously pumped into the reaction mixture using a single-channel peristaltic pump (LongerPump i150) with a constant flow rate of 0.30 ml/h. In batch experiments, 0.60 ml (equals to the total volume of solution pumped in 2 h using a flow rate of 0.30 ml/h) of solution of Na_2_SO_3_ and sulfuric acid [c(Na_2_SO_3_) = 2.0 M and c(H^+^) = 0.31 M] was added all at once to the initial reaction mixture prepared the same way as described in the semibatch experiments. For the ^1^H-NMR studies 60 μl of reaction mixture was sampled and the polymerization was quenched by the addition of 540 μl methanol-*d*_*4*_. The spectra of polymers were recorded on a 500 MHz Varian Unity/Inova 500B spectrometer. The final product was collected and dialyzed against DI water and freeze-dried for NMR and Gel permeation chromatography (GPC) studies. GPC analysis was conducted on an Agilent 1,260 system equipped with a refractive index detector, using DMF with 0.05 mol/L LiBr as an eluent, at a flow rate of 1.0 ml/min at 50°C. Monodispersed polystyrene (PS) standards (Agilent Technologies) with molecular weights ranging from 580 to 3.2 × 10^6^ Da were used to obtain the standard curve. Dynamic light scattering (DLS) analysis was conducted on a Delsa Nano C particle size and zeta potential analyzer (Beckman Coulter, Inc.) to determine the average hydrodynamic diameters (Ave. D.) and polydispersity indexes (PDI) at 15, 30, 60, 90 and 120 min. Morphology of the self-assembled polymersomes was observed under a field scanning electron microscope (FESEM, Supra 55VP) and/or transmission electron microscope (TEM, JEOL JEM-2100) in samples collected at 15, 60 and 120 min. Energy dispersive spectroscopy (EDS) analysis and elemental mapping were also conducted on FESEM (Supra 55VP).

### Calculation of Species Distribution of H_2_SO_3_

The species distribution of the H_2_SO_3_ as the function of pH was calculated by using the HYDRA/MEDUSA software package (Puigdomenech, 2015). The acid dissociation constants of H_2_SO_3_ are: pK_a1_ = 1.91 and pK_a2_ = 7.22. The total concentration of SO_3_^2-^ used for calculation was 0.8 mm, which was calculated by considering the approximate inflowed 2.0 M Na_2_SO_3_ in 10 min (flow rate of 0.30 ml/h), diluted to 20 ml. Except neutralization, no other reactions were considered.

## Results and Discussion

**SCHEME 1:**
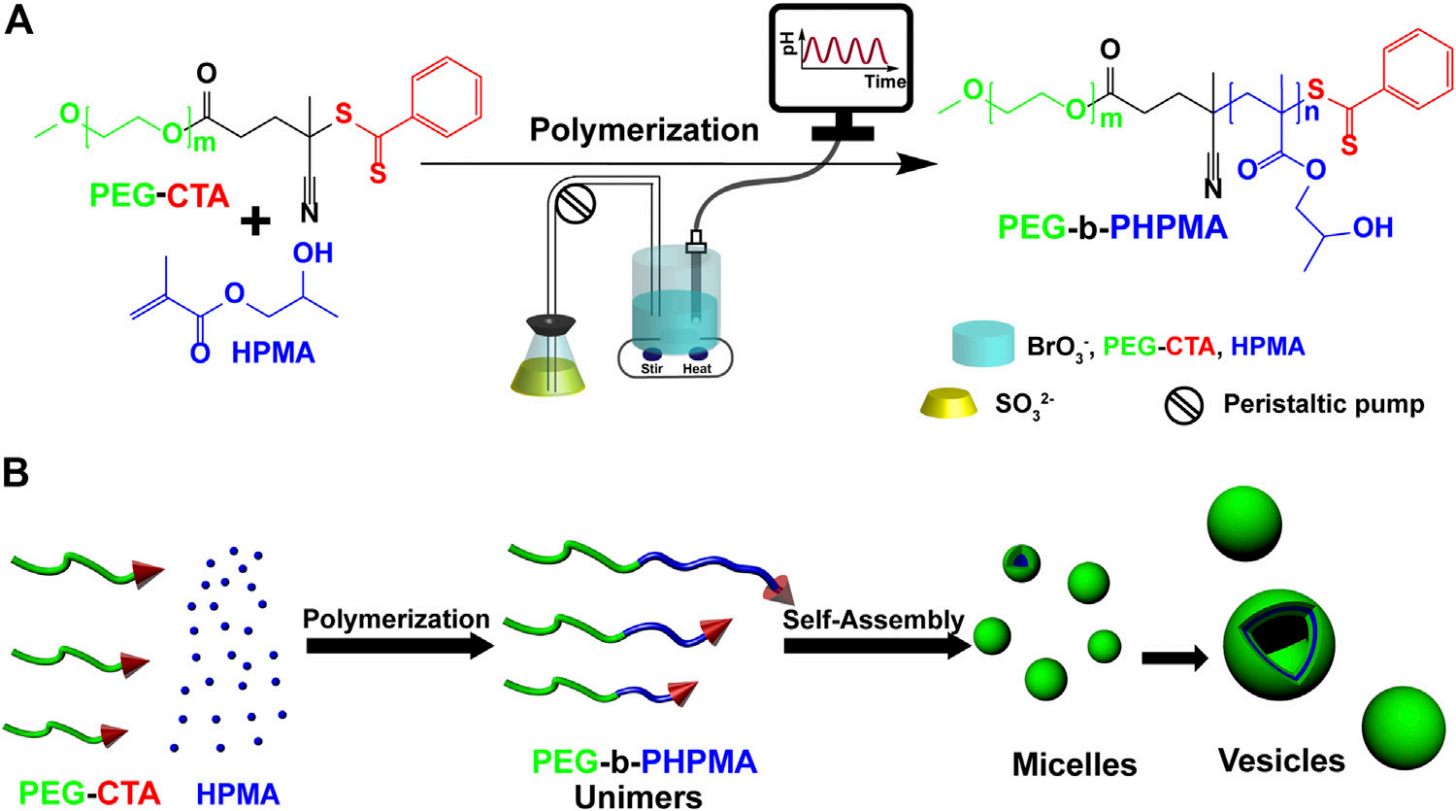
**(A)** Synthesis of an amphiphilic PEG-b-PHPMA block copolymer through polymerization driven by a semibatch BrO_3_^−^ − SO_3_^2–^ pH oscillator. **(B)** Induced self-assembly process.

Scheme 1 illustrates the main processes of the semibatch BrO_3_^−^−SO_3_^2–^ pH-oscillator driven PISA, during which the HPMA is polymerized on the poly(ethylene glycol) 4-cyano-4(phenylcarbonothoylthio)pentanoate (PEG-CTA) solvophilic macro-RAFT agent. The polymerization is initiated by the pH oscillator generated radicals (Scheme 1A). HPMA is a hydrophilic (water soluble) monomer that becomes hydrophobic after polymerization. The growth of the hydrophobic chain on the hydrophilic PEG-CTA results in the formation of an amphiphilic diblock copolymer (PEG-b-PHPMA), which self-assembles into micelles and eventually transforms to vesicles (Scheme 1B).

### Polymerization Initiated by the Semibatch BrO_3_^−^ − SO_3_^2–^ pH Oscillator

**FIGURE 1:**
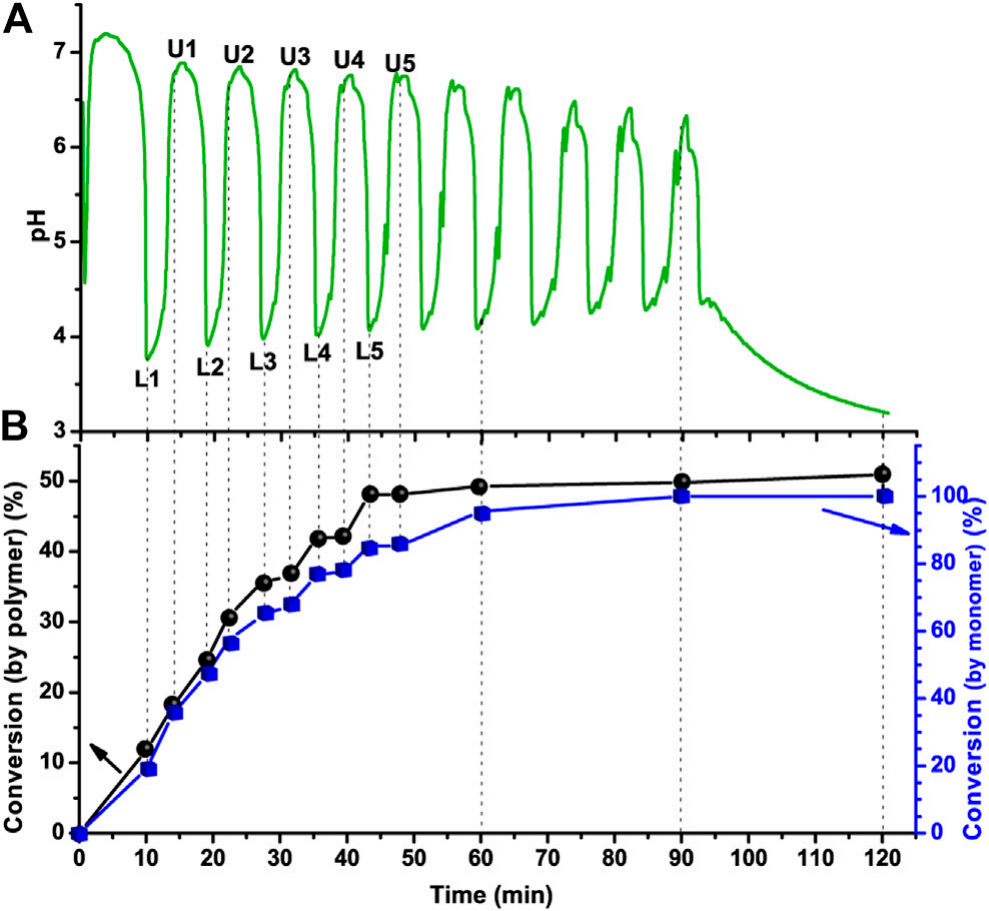
Polymerization driven by pH oscillatory chemistry **(A)** The pH oscillation curve in a semibatch BrO_3_^−^ − SO_3_^2–^ pH oscillator initiated system and **(B)** the corresponding monomer conversions (%) of PEG-b-PHPMA calculated by the increase in the area of polymer peak (-CH_2_-C(COOR)-CH_3_, 0.8–1.0 ppm, black line) or the decrease in the area of monomer peak (CH_2_ = , used the one at 6.1–6.2 ppm, blue line); the monomer conversions were determined by ^1^H-NMR spectra of the reaction mixtures sampled out at different times. The solution of sodium sulfite and sulfuric acid (c(Na_2_SO_3_) = 2.0 M and c(H^+^) = 0.31 M) was inflowed at a rate of 0.30 ml/h to the 20.0 ml solution of [NaBrO_3_]_0_ = 0.10 M, PEG-CTA (17.3 mg, 8 μmol) and HPMA (112.2 μl, 800 μmol, DP_target_ = 100), T = 40°C.

The recorded pH oscillations in the optimized semibatch BrO_3_^−^ − SO_3_^2–^ pH-oscillator driven PISA of PEG-b-PHPMA with different target degrees of polymerization (DP_targets_) of HPMA are shown in Figure 1A (DP_target_ = 100) and Supplementary Figure S1 (DP_target_ = 65 and 130). The change of period times, amplitudes, and the minimal pH values in each pH oscillation periods, at composition DP_target_ = 100 are shown in Supplementary Figure S2. The period time of the pH oscillations stabilized within a narrow range from 8 to 10 min as reaction proceeded, while the amplitudes (ΔpH) kept decreasing from 3.5 to 2 pH units, and the minimal pH values (pH_min_) increased from ∼3.75 to ∼4.25.

When polymerization was coupled with the pH oscillator, compared to the H^+^ concentration of the inflowed solution [c(Na_2_SO_3_) = 2.0 M and c(H^+^) = 0.06 M] in the pure BrO_3_^−^ − SO_3_^2–^ pH-oscillator (Supplementary Figure S3B), to maintain the original high amplitude pH oscillations, higher input of H^+^ (c(H^+^) = 0.31 M) was required. When this high concentration H^+^ (c(H^+^) = 0.31 M) was inflowed to the pure system, oscillations in the pH cannot be measured (Supplementary Figure S3A).

The conversion (%) of HPMA was determined by ^1^H-NMR. Reaction mixture samples were collected at different times (labeled on the pH oscillation curve in Figure 1A). Here LX/UX indicates the lower or upper point in the X oscillatory cycle. The monomer conversion (%) vs. time (Figure 1B) was calculated two ways, from the decrease in the area of the monomer peak and from the increase in the area of the polymer peak in the ^1^H-NMR spectra (Supplementary Figure S4). The final monomer conversion at 120 min calculated in from the increase in the area of polymer peak was 51% (Supplementary Table S1), while based on the decrease of the area of the monomer peak, the final conversion at 120 min was calculated to be 100%. Monomer evaporation (T = 40°C) was the potential reason for the difference in the conversions calculated in the two different ways.

**FIGURE 2:**
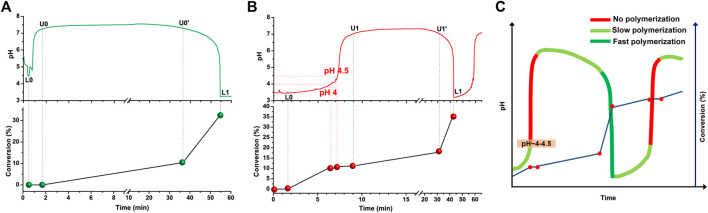
pH oscillations and corresponding monomer conversions in the first oscillatory period. The starting pH value (after the addition of PEG-CTA and HPMA) **(A)** 4.5 or **(B)** < 4 (lowered by the addition of H_2_SO_4_). The other conditions are the same as in Figure 1, except the T = 25°C **(C)** Periodic changes in the rate of polymerization in pH ranges of each period.

An interesting, and expected, behavior consisting of a series of stepwise increases in monomer conversions (%) vs. time, is observed and indicates the presence of periodic polymerization in our system (Figure 1B). The two-way oxidation of SO_3_^2–^ by BrO_3_^−^ is known to be the source of the pH oscillations in the BrO_3_^−^ − SO_3_^2–^ pH-oscillator (Szántó and Rábai, 2005). The complete oxidation of HSO_3_^−^ and H_2_SO_3_ to SO_4_^2–^ produces H^+^ autocatalytically and provides a positive feedback (Table 1, R3 and R4). The partial oxidation of HSO_3_^−^ to the relatively stable intermediate, S_2_O_6_^2–^ (Table 1, R5), consumes H^+^ which is the source of the delayed negative feedback in the pH oscillatory cycle with the contribution of the protonation of the inflowed SO_3_^2–^. Possible radical formation and consumption reactions are listed in Table 1 (R6–R11). The SO_3_^−^*, HSO_3_*, OH* are potential initiators (Mukherjee et al., 1966). Halogen atom endgroup has not been found in BrO_3_^−^/SO_3_^2–^ redox pair-initiated polymerization (Mukherjee et al., 1966). Both the positive (R3, R4) and negative feed-back processes (R5) involve free radical steps (R6, R7). A considerable difference in the amount of produced free radicals is expected during the H^+^ producing and consuming reactions, since the major route for the oxidation of the sulfite is known to be the complete oxidation, while only 1–2% of the initial SO_3_^2–^ is oxidized to S_2_O_6_^2–^. Our experiments completely support this. The polymerization is found to be faster as the pH decreases and slower, or negligible, when the pH increases (especially so after the first three periods of oscillation). To further characterize this system, a series of experiments were conducted at different starting pH values and at different temperatures (Figure 2 Supplementary FigureS5). Working at a lower temperature (T = 25°C) increases the period time of the oscillations and allowed us to perform more accurate sampling. Figures 2A, B show the pH change and the corresponding conversion (%) in time at a DP_target_ = 100 at 25°C at different initial pH values. When premixed PEG-CTA and HPMA were added to the bromate solution, without prior adjustment of the initial pH, the pH of the system immediately dropped from ∼5.5 (pH of 0.10 M NaBrO_3_) to ∼4.5 (Figure 2A). Then, the pH quickly increased to >7.0 as the sulfite solution (Na_2_SO_3_ + H_2_SO_4_) was continuously inflowed at a steady flow rate (0.30 ml/h). Polymerization was not detected between the points L0 and U0. At point U0, the slow oxidization of HSO_3_^−^ by the bromate ion is triggered and slowly produces H^+^ (R3). This reaction generates initiating radicals for the polymerization between U0 and U0′ indicated by the slow increase in the conversion (%). The increasing H^+^ concentration increases the concentration of the more reactive H_2_SO_3_ and R4 reaction is activated. The rate of R4 is much higher than the rate of R3. As the overall reaction rate increases, the concentration of free radicals also increases, which is clearly accompanied by an increment in the rate of polymerization between the U0′ and L1 points.

**TABLE 1:** The elementary reactions of the pure B-S pH oscillator (reproduced from Szántó and Rábai, 2005).

	Reactions	Rate constants (T = 45°C)
**BrO**_**3**_^**−**^ **− SO**_**3**_^**2–**^ **pH-oscillator**		
R1	SO_3_^2– ^+ H^+^ ↔ HSO_3_^−^	k_1_ = 2.0 × 10^10^ M^−1^s^−1^ k_−1_ = 2.0 × 10^3^ M^−1^s^−1^
R2	HSO_3_^− ^+ H^+^ ↔ H_2_SO_3_	k_2_ = 12.0 × 10^9^ M^−1^s^−1^ k_−2_ = 2.0 × 10^8^ M^−1^s^−1^
R3	3 HSO_3_^− ^+ BrO_3_^−^ → 3 SO_4_^2–- ^+ Br^− ^+ 3 H^+^	k_3_ = 0.13 M^−1^s^−1^
R4	3 H_2_SO_3_ + BrO_3_^−^ → 3 SO_4_^2– ^+ Br^− ^+ 6 H^+^	k_4_ = 30 M^−1^s^−1^
R5	6 H_2_SO_3_ + BrO_3_^−^ → 3 S_2_O_6_^2– ^+ Br^− ^+ 3H_2_O + 6 H^+^	k_5_ = 2 M^−1^s^−1^
**Radical formation**		
R6	BrO_3_^−^ + HSO_3_^−^ → BrO_2_^−^ + SO_3_^‒^* + OH*	
R7	BrO_3_^−^ + H_2_SO_3_ → BrO_2_^−^ + HSO_3_* + OH*	
**Radical consumption**		
R8	2 HSO_3_* → H_2_S_2_O_6_	
R9	SO_3_^‒^* + OH* → SO_4_^2‒^ + H^+^	
R10	HSO_3_* + OH* → SO_4_^2‒^ + 2H^+^	
R11	I* → P_n_*	

Proposed radical formation and consumption reactions. M = monomer, P_n_* = polymer radical. $I^{*}\overset{nM}{\to }P_{n}^{*}$ represents the polymerization process.

Figure 2B shows the pH and the conversion (%) in time when the initial pH value of the bromate solution was set to pH∼3.5 (by adding H_2_SO_4_ to the solution of bromate, PEG-CTA and HPMA). The pH of the reaction mixture started to increase as the sulfite solution (Na_2_SO_3_ + H_2_SO_4_) was inflowed but in a much slower rate, than in the previous case and slow polymerization was detected immediately from point L0 initiated by the radicals generated in R7. After the system’s pH reaches a critical value (pH = 4–4.5) the polymerization was temporarily interrupted, indicating that the increase in pH here is predominantly caused by the protonation of the continuously inflowed sulfite, and the radical generation is negligible until point U1 is reached [Note that, the calculated species distribution of H_2_SO_3_-HSO_3_^−^-SO_3_^2−^ vs. pH (Supplementary Figure S7) shows a similar pH value of ∼4.5, above which H_2_SO_3_ is no longer available.] Then during the pH drop phase the polymerization is initiated, and from point U1 to U1′ its rate was found to be relatively slower and from U1′ to L1 faster [An experiment at T = 40°C with the adjustment of the starting pH to 3.8 was also run and, similarly, stepwise increases in the conversion (%) were detected (Supplementary Figures S5, S6)].

Following the conversion (%) in time for the complete working pH range of the pH oscillator, let us not only identify the ranges of each period when polymerization takes place in the system, but also to determine the relatively faster, slower and negligibly slow phases, providing proof of our assumption for the periodic radical formation and polymerization (Figure 2C). The formation of the initiating radicals (SO_3_^−^*, HSO_3_*, and OH*) is switched ON and OFF by the BrO_3_^−^ − SO_3_^2–^ pH oscillator. This is in contrast with B-Z-assisted periodic polymerization, in which the initiating malonyl radicals are always available and periodic termination by BrO_2_* occurs (Pojman et al., 1992; Washington et al., 1999).

The DP of the purified final PEG-b-PHPMA product was also determined by ^1^H-NMR (DP = 54, Supplementary Figure S8A) and GPC (DP = 46, Supplementary Figure S8B and Supplementary Table S1), the latter is close to the conversion calculated from the ^1^H-NMR spectrum of the final reaction mixture sampled at 120 min (DP = 51, Figure 1B, Supplementary Figure S4 and Supplementary Table S1). The dispersity (Đ) of the final PEG-b-PHPMA was calculated to be 1.97 using GPC (Supplementary Table S1).

### Polymerization in Non-oscillatory Regimes

**FIGURE 3:**
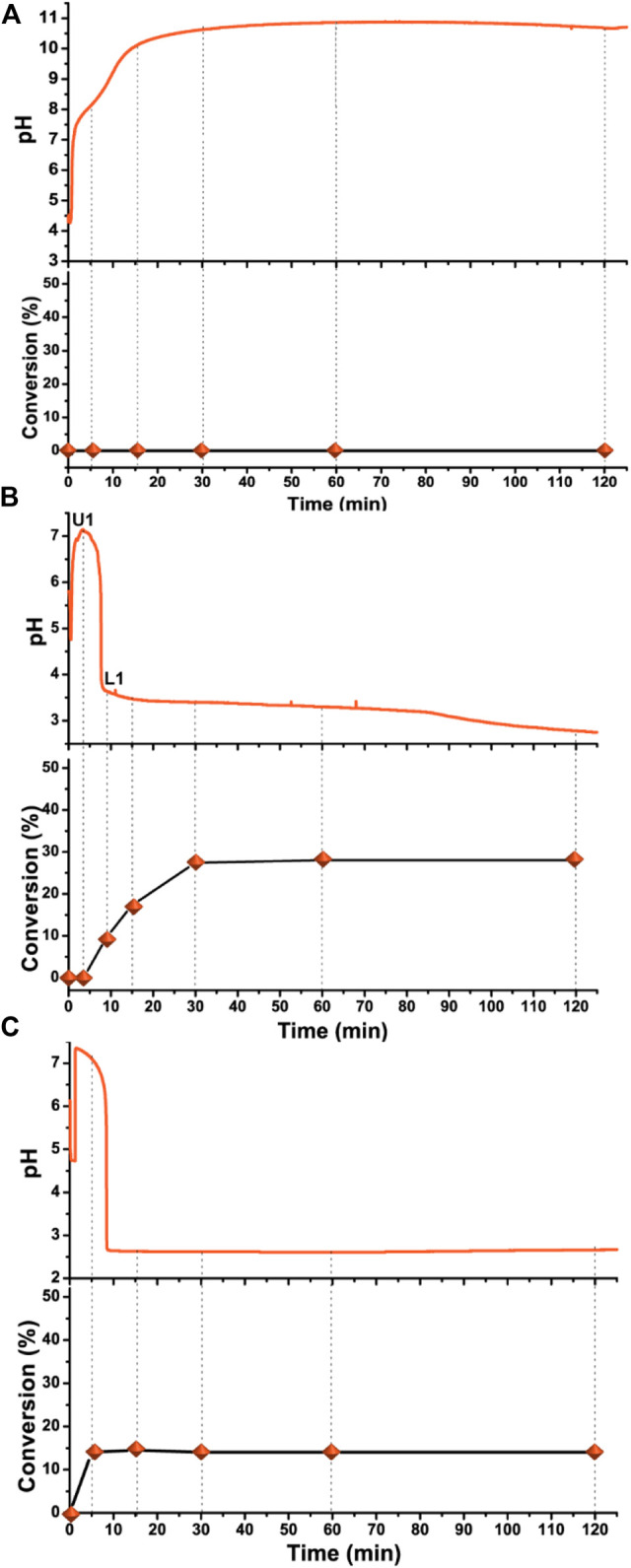
pH changes and corresponding monomer conversions in non-oscillatory regimes in semibatch reactor and in batch reactor. The H^+^ concentration in the inflowed sulfite solution are **(A)** 0.21 M or **(B)** 0.50 M; all the other conditions are the same as in Figure 1 **(C)** In batch reactor (0.60 ml c(Na_2_SO_3_) = 2.0 M and c(H^+^) = 0.31 M solution was added in the beginning all at once, instead of flowing in, the other conditions are the same as in Figure 1.

The pH and monomer conversions vs. time curves recorded in experiments when the system was in its non-oscillatory regime (Figure 3). The higher or lower input H^+^ concentrations make the system operate in a low or high pH steady state (Figures 3A, B). Figure 3A shows the pH change curve, and corresponding monomer conversion curve, when the inflowed solution had a lower H^+^ concentration [c(H^+^) = 0.21 M]. R3 and R4 (Table 1) could not be triggered, and the pH value kept growing until it was close to the pH of the inflowed solution. No polymerization was observed during the experiment because the oxidation of the unprotonated SO_3_^2‒^ by BrO_3_^−^ is negligibly slow (Figure 3A and Supplementary Figure S9A). However, some reactions, such as disproportionation reactions and ester bond cleavage of monomers, may have occurred, especially in highly basic conditions (pH > 10), as can be inferred from the complicated structure of the -CH(OH)-CH_3_ peak at ∼1.2 ppm and the decrease in the height of double bond peaks at 5.7 and 6.2 ppm shown in the ^1^H-NMR spectra (Supplementary Figure S9A). A higher H^+^ input (c(H^+^) = 0.50 M) can also bring the system out of the oscillatory regime. The pH of the system increased quickly once sulfite solution was continuously pumped in. Then the autocatalytic oxidation of sulfite by bromate brought the pH quickly down to a value of pH < 4 and the pH stayed at the acidic steady state (Figure 3B). Although oscillations did not occur, radical formation was not stopped due to the ongoing R3, R4 and R5, when the pH < 4. Most of the polymerization happened during the pH drop phase in the first 30 min. The final monomer conversion at 120 min was much lower (<30%) than in the oscillatory regime (Supplementary Figure S9B). Experiments under the same conditions as in the original semibatch reactor (Figure 1) were also conducted in a batch reactor (Figure 3C), when a 0.60 ml solution (the same volume inflowed in 2 h to a semibatch reactor) of sulfite and H^+^ [c(Na_2_SO_3_) = 2.0 M and c(H^+^) = 0.31 M] was added all at once, the pH quickly dropped to an acidic state, and polymerization stopped once all the sulfite was consumed and the conversion was much lower, < 15% (Figure 3C and Supplementary Figures S9C).

### Self-Assembly of the PEG-b-PHPMA Diblock Copolymers

**FIGURE 4:**
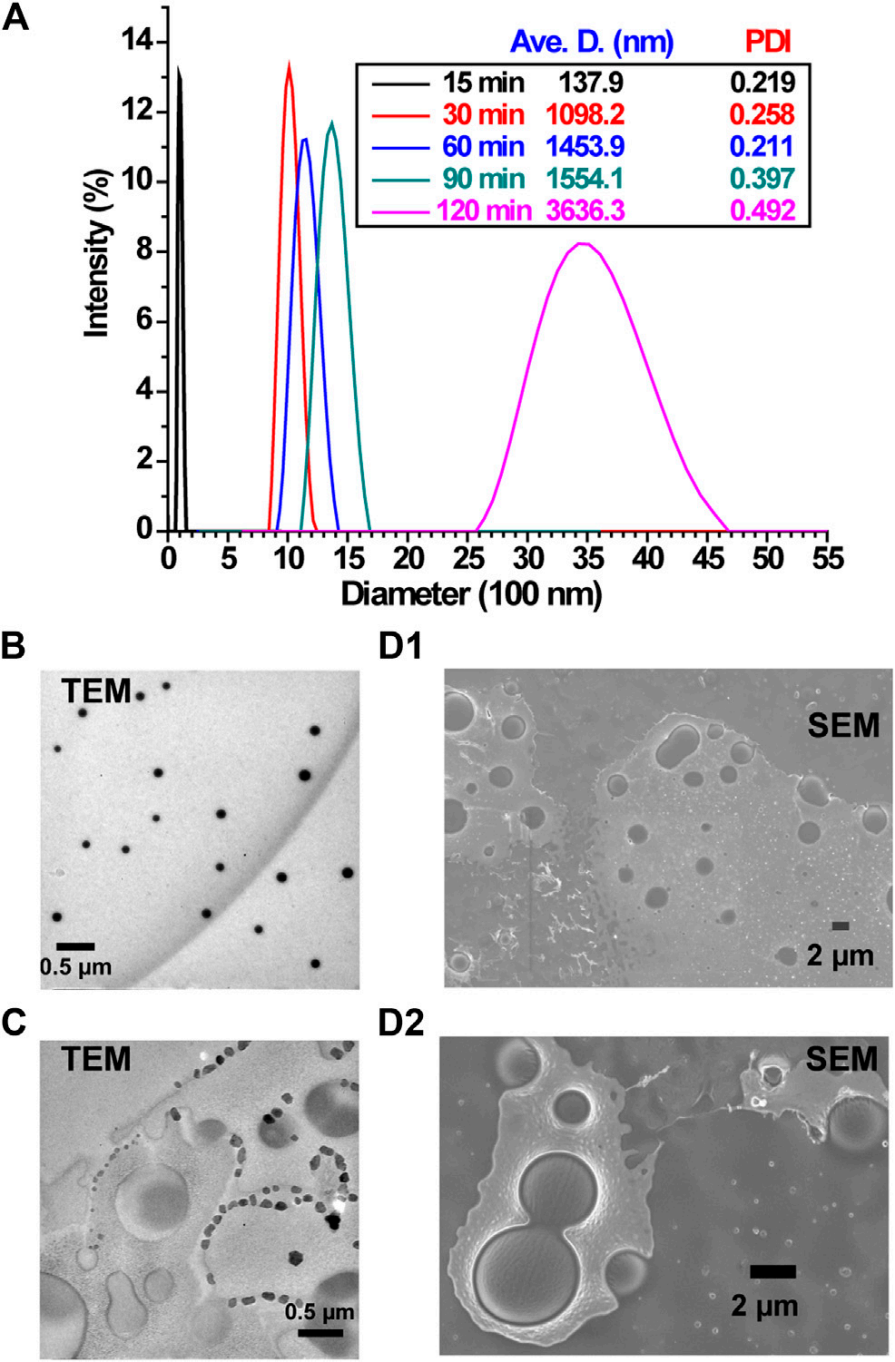
Size and morphology of PEG-b-PHPMA particles: **(A)** dynamic light scattering (DLS) data and T/SEM imaging of samples taken at different times [**(B)** 15 min, TEM; **(C)** 60 min, TEM **(D1)**, **(D2)** (zoomed in) 120 min, SEM].

Finally, the dynamical evolution as a collective system of the self-assembled objects generated during polymerization induced self-assembly driven by pH oscillatory chemistry was studied by monitoring the size and morphology changes of the generated self-assembled structures using DLS and SEM/TEM respectively (Figure 4) (The reaction conditions were the same as in Figure 1.) Figure 4A shows the change in the average size of self-assembled aggregates from ∼138 nm at 15 min to 1–1.5 μm at 60 min, and to ∼5 μm at 120 min. The formed vesicles were stable, but some precipitation was observed after 2–3 days.

PEG-b-PHPMA diblock copolymers, consisting of similar length of the stabilizing hydrophilic PEG-block and similar length of the hydrophobic core forming PHPMA blocks, as our final polymer, but synthesized by light-induced initiation has been reported to form only nanoscale spheres (Ren and Perez-Mercader, 2017). The essential presence of high concentration salt in the BrO_3_^−^ − SO_3_^2–^ pH oscillator, especially the continuously supplied Na_2_SO_3_ and the produced Na_2_SO_4_, can contribute to giant vesicle formation of PEG-b-PHPMA synthesized by pH oscillatory chemistry (Figures 4D1, D2). The increasing ionic strength makes entropic forces become more favorable to exclude hydrophobic structures, resulting in a shift toward higher order morphologies (Zhou et al., 2017). The solubility of hydroxyl group containing polymers, such as poly(vinyl alcohol) (PVA), has been reported to be greatly reduced by the addition of salt, and low concentrations of Na_2_SO_4_ serves as a precipitant for PVA (Gupta et al., 2012). The turbidities of purified PEG_43_-b-PHPMA_51_ suspensions were seen to gradually increase from pure water to NaBrO_3_ solution and to the mixed solution of NaBrO_3_ + Na_2_SO_3_ + H_2_SO_4_ (Supplementary Figure S10). The gradual increase in salt concentration can change the volume fraction of the hydrophobic tail and can lead to a higher packing parameter, which promotes the formation of giant vesicles (Scheme 1B and Figure 4). Vesicle budding and division were also seen in both TEM (Figure 4C) and SEM (Figures 4D1, D2) images. A possible reason for this evolution is the autonomously generated chemical gradient through the membrane by the continuously increasing salt concentration and by the difference in chemical reactions outside of the vesicles and entrapped by the vesicles.

Beside micelles and vesicles, other type of intermediate morphologies, such as worms and jellyfish, oligolamellar vesicles have been reported previously in the polymerization-induced self-assembly of HPMA using PEG macro-CTA at a very narrow range of the phase diagrams (Warren et al., 2014). Such kind of self-assembled objects were not observed during the pH oscillator-driven PISA of PEG-b-PHPMA. The reason of the absence of these morphologies could be the lower level of control on the degree of the polymerization and the higher dispersity of the resulting copolymer.

As indicated by an energy dispersive spectroscopy analysis (EDS), the observed objects in the 120-min sample contain the element carbon, thus proving that they are made of polymer (Supplementary Figure S11).

## Conclusion

In summary, radicals generated in the semibatch BrO_3_^−^ − SO_3_^2–^ pH oscillator can be used as chemical fuel and engine to drive polymerization induced self-assembly for the autonomous one-pot synthesis of functional giant vesicles. Importantly, radical formation is found to be forced ON and OFF by the pH oscillator, inducing a periodic polymerization. The detailed study of the polymer conversion (%) as the function of time let us determine the changes in the relative rate of polymerization in different pH regimes. The changes in the rate of polymerization and its temporal interruption correlate with the expected dominating chemical reactions and their reaction rates during one oscillatory period. The periodic polymerization indicates the presence of the same free radicals in the BrO_3_^−^ − SO_3_^2–^ pH oscillator as in the conventional redox initiation by the BrO_3_^−^/SO_3_^2–^ redox pair, but the different kinetics has a great effect on the final conversion (%) in a particular composition.

The high salt concentration in the BrO_3_^−^ − SO_3_^2–^ pH oscillator, as an additional control parameter, effects the morphology of the self-assembled block copolymer and contributes to the formation of microscale self-assembled structures.

The understanding of the periodic initiation of the polymerization by a pH oscillator helps the extension of oscillatory chemistry-initiated systems to other block copolymers and to other groups of oscillatory chemical reactions. For example the Cu(II) catalyzed S_2_O_8_^2–^−S_2_O_3_^2–^ system is a good candidate (Orbán and Epstein, 1989), hence the redox reaction between K_2_S_2_O_8_ and Na_2_S_2_O_3_ has been used before as an initiator in RAFT polymerization (Bai et al., 2008; Zheng et al., 2008; Li et al., 2009). Our system paves the way for applications in fields like origin of life (Silva and Lazcano, 2004) in geochemical settings, where changes in pH are induced by complex geochemical phenomena, or for the introduction of pH sensitive factors integrated within the amphiphilic block copolymers. pH oscillator driven PISA offers a practical application potential for the autonomous generation of active/functional materials, where pH oscillation-controlled morphology transformations of polymersomes are needed in other contexts requiring regulation, as in the programmable release of cargo molecules Isakova and Novakovic 2017.

## Data Availability

The original contributions presented in the study are included in the article/Supplementary Material, further inquiries can be directed to the corresponding authors.
