# Supplementary material for: Periodic Polymerization and the Generation of Polymer Giant Vesicles Autonomously Driven by pH Oscillatory Chemistry
Source: Front Chem. 2021 Feb 22;9:576349. doi: 10.3389/fchem.2021.576349 (PMC7992010; doi:10.3389/fchem.2021.576349)
Supplement: Supplementary file 1 [file table1.docx]

Supplementary Material

# Supplementary Figures and Tables

**Table S1.** Molecular weights and Dispersity (*Đ*) of PEG, PEG-CTA and PEG-b-PHPMA (DP_target_ = 100, polymerized for 2 hours) determined by ^1^H-NMR and GPC.

| **Polymer** | **M_n_-NMR (Da)** | **M_n_-GPC (Da)** | **M_w_-GPC (Da)** | ***Đ*** | **DP of HPMA** |
| --- | --- | --- | --- | --- | --- |
| PEG^a^ | 1953^b^ | 2300 | 2700 | 1.17 | 0 |
| PEG-CTA | 2230^c^ | 2500 | 3400 | 1.36 | 0 |
| PEG-b-PHPMA | 9568 | 9200 | 18100 | 1.97 | 50.9^d^/46.3^e^ |

^a^The mPEG with a molecular weight of 1900Da (provided by the company) was purchased from Fluka; ^b^Calculated by the integration ratio between -C**H**_2_C**H**_2_O- (3.59-3.68 ppm) and terminal –C**H**_3_ (~3.35ppm) group; ^c^The CTA modification efficiency, determined by the integration ratio between -C**H**_2_- of the terminal of mPEG that near the newly formed ester group (mPEG-O-CH_2_-C**H**_2_-OCO-CTA, ~4.15 ppm) and the terminal –C**H**_3_ of mPEG (~3.35ppm), was 99.3%, so the M_n_ of PEG-CTA determined by NMR is 2230 Da; ^d^Determined by NMR of the final reaction mixture; ^e^Determined by GPC.


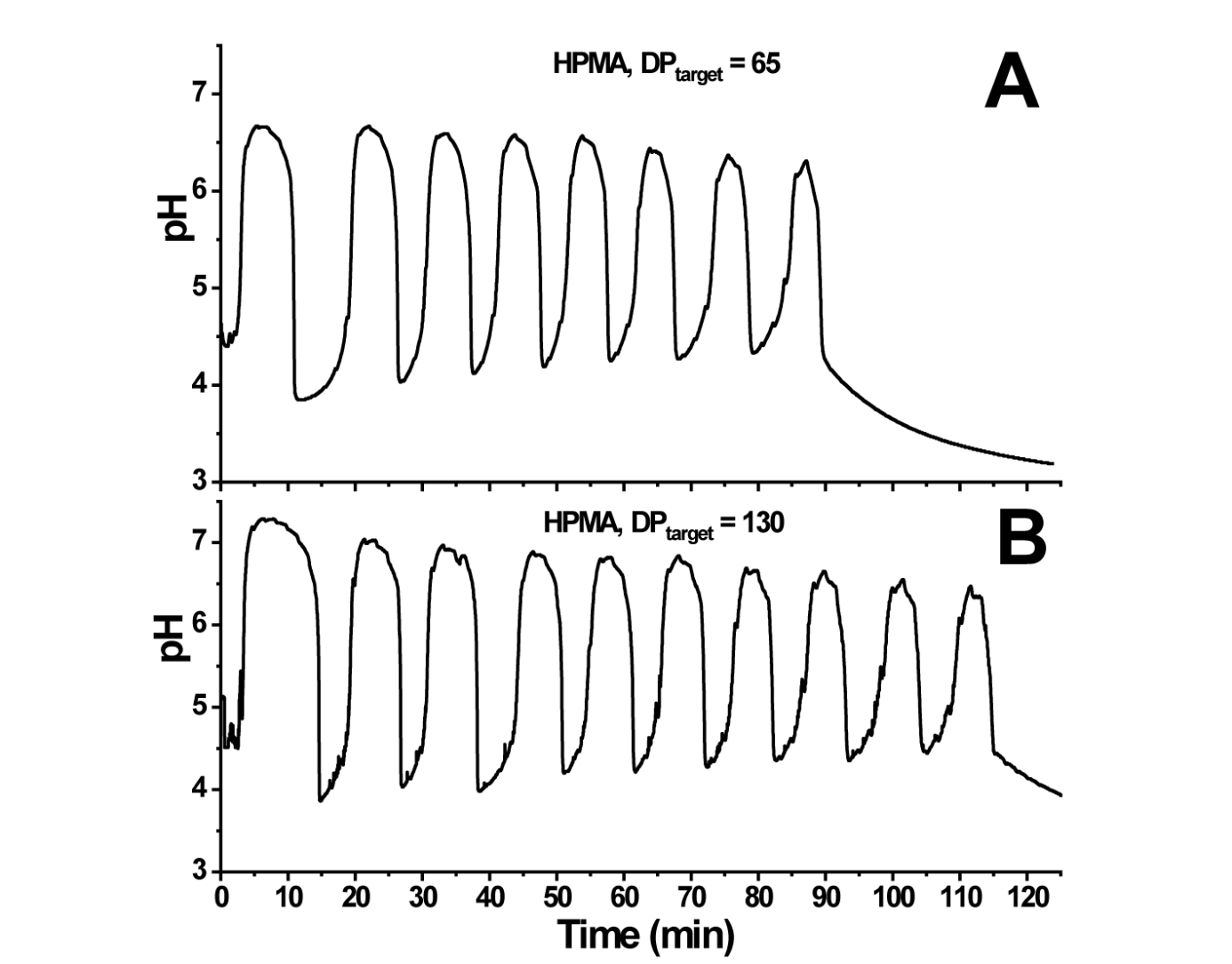


**Figure S1.** pH oscillation curves of pH-O-PISA of PEG-b-PHPMA with a HPMA DP_target_ = 65 (**A**) and 130 (**B**). Reaction conditions: (**A**) 2.0 M Na_2_SO_3_ + 0.31 M H^+^ (H_2_SO_4_) solution inflowed at a of 0.03 mL/hour to the 20.0 mL solution of PEG-CTA 17.3 mg (8 μmol), HPMA 73.0 uL (DP_target_ = 65), 0.1 M NaBrO_3_; (**B**) 2.0 M Na_2_SO_3_ + 0.31 M H^+^ (H_2_SO_4_) solution inflowed at a of 0.03 mL/hour to the 20.0 mL solution of PEG-CTA 17.3 mg (8 μmol), HPMA 145.8 uL (DP_target_ = 130) and 0.1 M NaBrO_3_, at T=40 ^o^C.


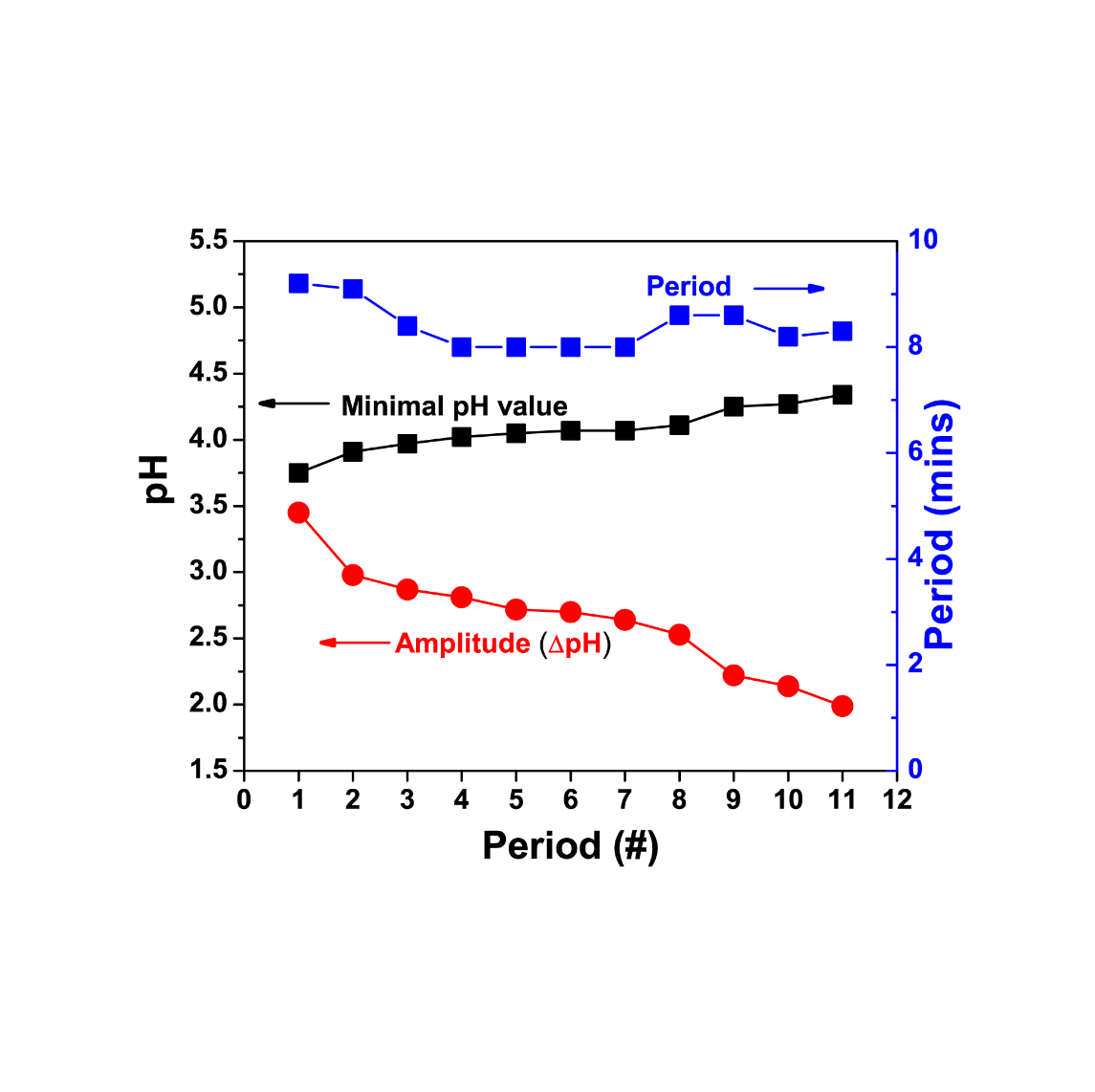


**Figure S2.** Changes in the period time (blue squares), amplitudes (red circles), and minimal pH values (black squares) of different periods. The solution of sodium sulfite and sulfuric acid (c(Na_2_SO_3_)= 2.0 M and c(H^+^)=0.31 M) was inflowed at a rate of 0.30 mL/hour to the 20.0mL solution of [NaBrO_3_]_0_ =0.10 M, PEG-CTA (17.3 mg, 8 μmol) and HPMA (112.2 μL, 800 μmol, (DP_target_ = 100)), T=40 °C.


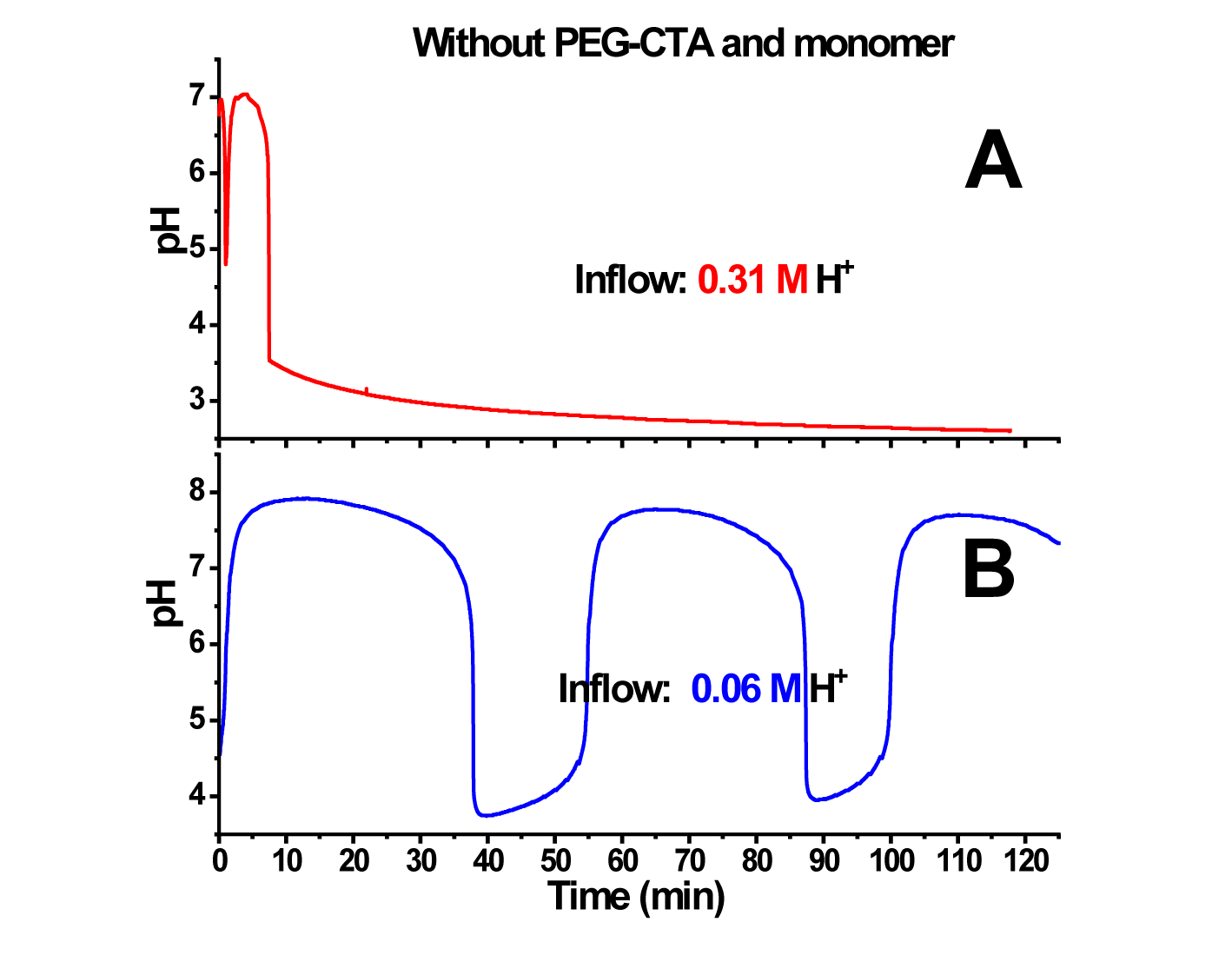


**Figure S3**. Without PEG-CTA and monomer, pH cannot oscillate for the same composition of Figure 1A (0.31 M H^+^ (H_2_SO_4_) in inflowed solution (**A**), much lower H^+^ (H_2_SO_4_) concentration in the inflowed solution (e.g. 0.06 M) are needed to restore pH oscillations for pure B-S recipe (**B**).


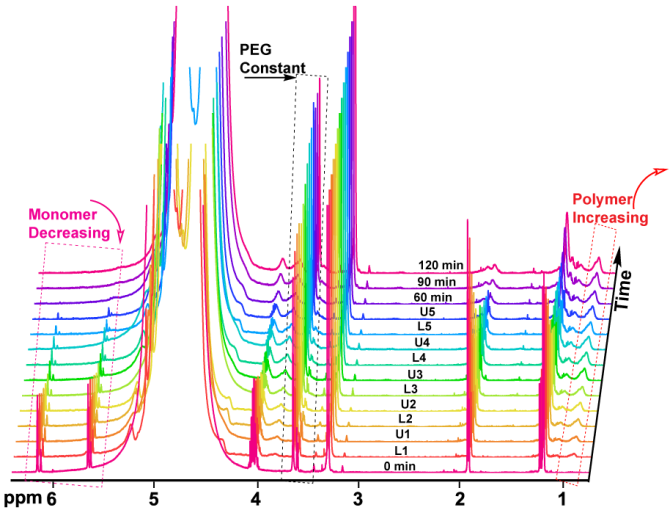


**Figure S4**. The ^1^H-NMR spectra of the reaction mixtures sampled out at different times (labelled on Figure 1A) for pH-O-PISA of PEG-b-PHPMA (DP_target_ = 100) at 40 °C (Reaction conditions are shown in Figure 1A).


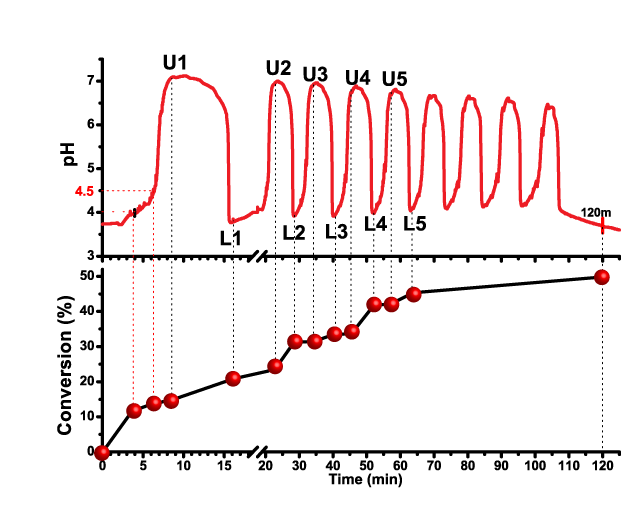


**Figure S5**. The pH oscillations and corresponding monomer conversion curves with the first period zoomed in for pH-O-PISA of PEG-b-PHPMA (DP_target_ = 100) at 40 °C, initial pH set to 3.8 (Reaction conditions are shown in Figure 1.).





**Figure S6**. ^1^H-NMR spectra for kinetic study for pH-O-PISA of PEG-b-PHPMA (DP_target_ =100) when 40 µL of 0.1 M H^+^ (H_2_SO_4_) was added in the beginning. NMR samples were prepared by dissolving 60 µL of the reaction mixture into 540 µL methanol-*d_4_*.


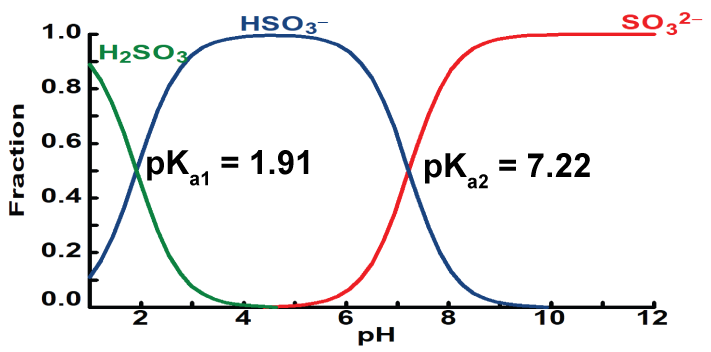


**Figure S7**. Calculated distribution of H_2_SO_3_‒ HSO_3_^–^ ‒ SO_3_^2–^ as a function of pH from 1 to 12.


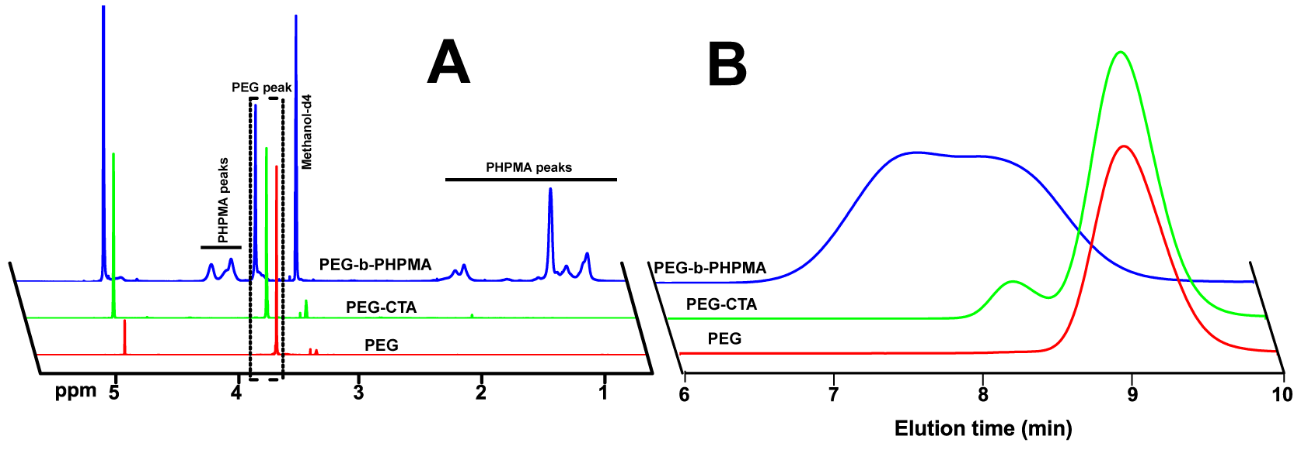


**Figure S8**. The ^1^H-NMR spectra (**A**) and the GPC curves (**B**) of PEG (methoxy poly(ethylene glycol), mPEG, 1900 Da, DP~43), PEG-CTA, and purified PEG-b-PHPMA (synthesized by pH-O-PISA with a DP_target_ = 100, reacted for 2 hours, purified by dialysis and freeze-drying).


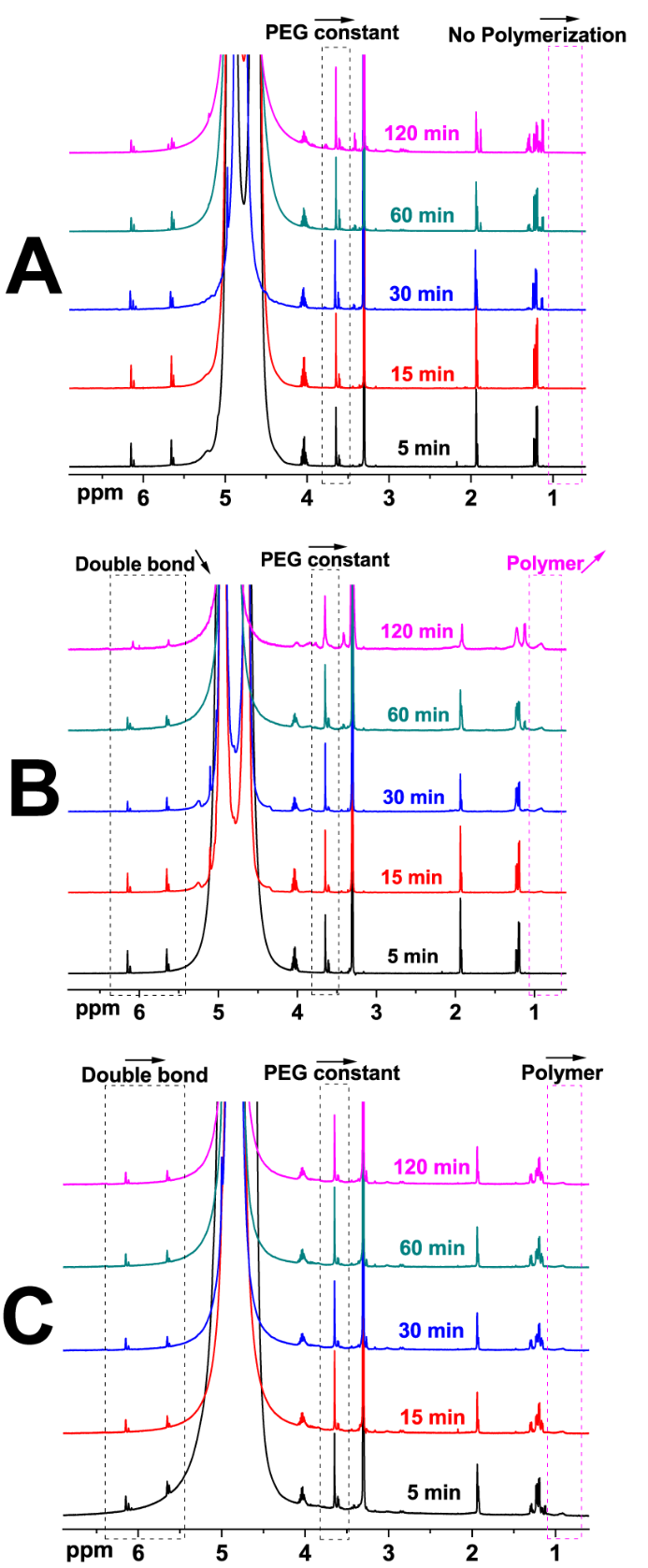


**Figure S9**. ^1^H-NMR spectra for kinetic study for polymerization of PEG-b-PHPMA at non-oscillatory regimes: semibatch (Inflow: 0.21 M H^+^ (**A**), 0.50 M H^+^ (**B**); batch (**C**).


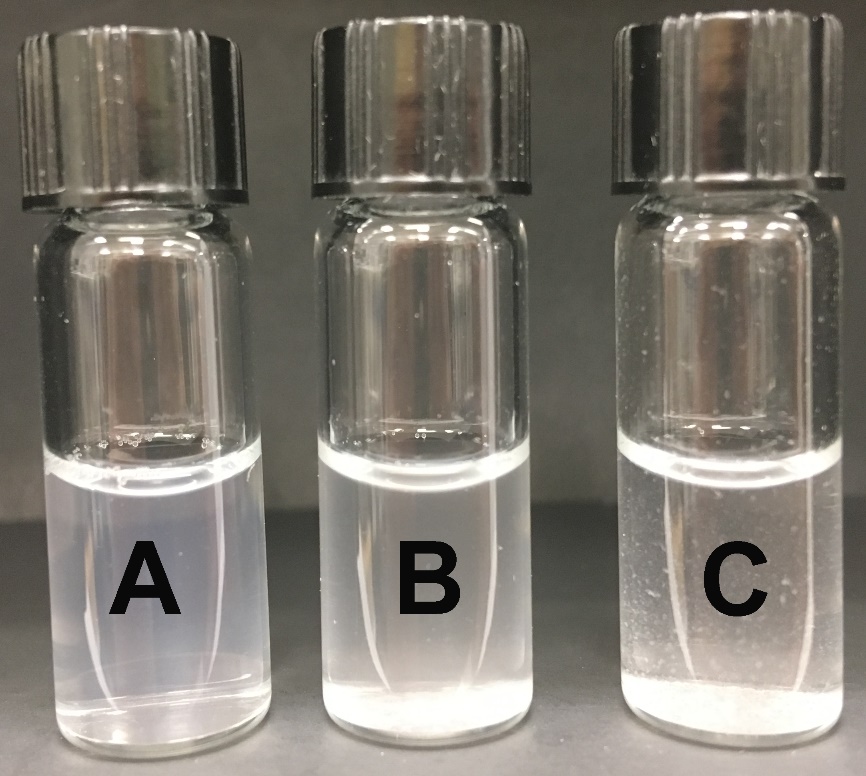


**Figure S10.** PEG-b-PHPMA copolymer (purified by dialysis and freeze-drying) dispersed in ultrapure water (**A**), 0.1 M NaBrO_3_ (**B**), and 0.1 M NaBrO_3_ + Na_2_SO_3_ + H_2_SO_4_ (**C**, to mimic the conditions after 2 hours reaction, solvent was prepared by adding 0.60 mL mixed solution of 2.0 M Na_2_SO_3_ + 0.31 M H^+^ (H_2_SO_4_) into 20 mL 0.1 M NaBrO_3_). (Solid content of PEG-b-PHPMA was 0.6 wt%.)


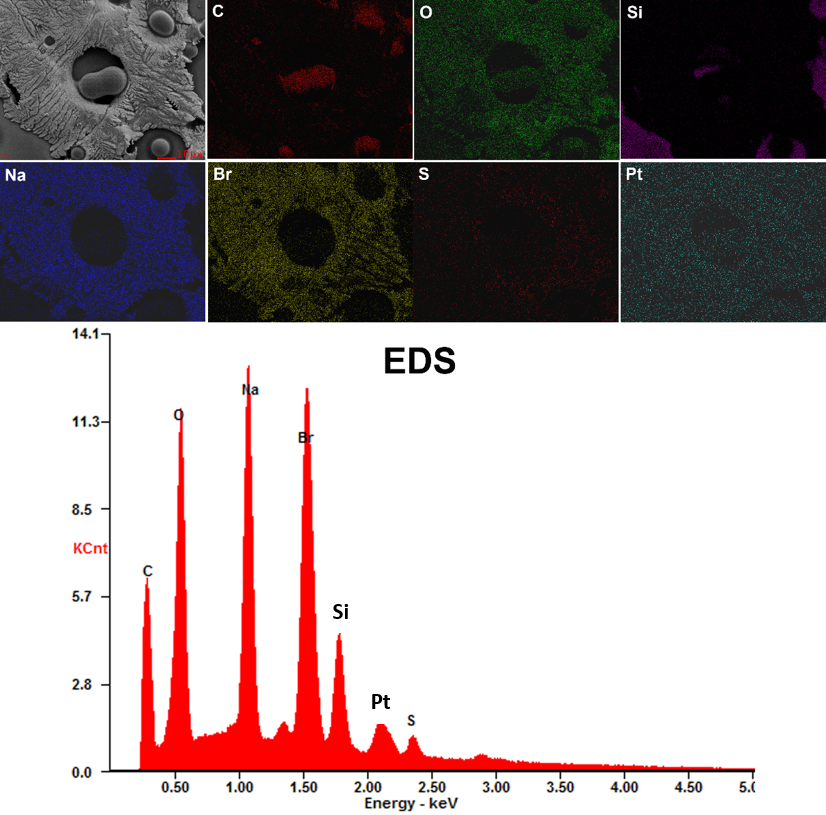


**Figure S11.** EDS mapping for the 120 min sample of PEG-b-PHPMA (unwashed) on possible elements in polymer and salts (C, O, Na, Br, S).
